# Short-Term Effects of Dupilumab in Eosinophilic COPD

**DOI:** 10.3390/jcm15020775

**Published:** 2026-01-18

**Authors:** Chiara Lupia, Daniela Pastore, Giuseppina Marrazzo, Giada Procopio, Antonio Giacalone, Federica Marrelli, Mariarosanna De Fina, Adele Emanuela De Francesco, Alessandro Vatrella, Santi Nolasco, Raffaele Campisi, Nunzio Crimi, Claudia Crimi, Girolamo Pelaia, Corrado Pelaia

**Affiliations:** 1 Department of Health Sciences, University “Magna Graecia” of Catanzaro, 88100 Catanzaro, Italy; chiaralupia1996@gmail.com (C.L.); danielapastore11@gmail.com (D.P.); giusimarrazzo@gmail.com (G.M.); giadapas42@gmail.com (G.P.); antonio.giacalone@live.it (A.G.); federicamarrelli1@gmail.com (F.M.); pelaia@unicz.it (G.P.); 2 Pharmacy Unit, AOU Renato Dulbecco, 88100 Catanzaro, Italy; mrsdefina@gmail.com (M.D.F.); adele.defrancesco@materdomniaou.it (A.E.D.F.); 3 Department of Medicine, Surgery and Dentistry, University of Salerno, 84084 Salerno, Italy; avatrella@unisa.it; 4 Department of Clinical and Experimental Medicine, University of Catania, 95123 Catania, Italy; nolascos@hotmail.it (S.N.); raffaelemd@hotmail.it (R.C.); crimi@unict.it (N.C.); claudia.crimi@unict.it (C.C.); 5 Department of Medical and Surgical Sciences, University “Magna Graecia” of Catanzaro, 88100 Catanzaro, Italy

**Keywords:** chronic obstructive pulmonary disease, eosinophilic COPD, dupilumab, type 2 inflammation, clinical practice

## Abstract

**Background/Objectives:** Patients with eosinophilic chronic obstructive pulmonary disease (COPD) often remain symptomatic despite optimized triple inhaled therapy. Dupilumab is a fully human monoclonal antibody that blocks the IL-4 receptor alpha subunit, thereby inhibiting IL-4 and IL-13 signaling. Evidence from randomized trials supports dupilumab for add-on treatment of type 2-high COPD, but data referring to short-term effectiveness in clinical practice are quite limited. **Methods:** We conducted an observational, compassionate-use study enrolling 13 consecutive outpatients with eosinophilic COPD (blood eosinophils ≥ 300 cells/µL) receiving add-on biologic therapy with dupilumab 300 mg every two weeks. Clinical (CAT, mMRC), functional (spirometry and body plethysmography), and inflammatory parameters (blood eosinophils/basophils, fibrinogen, FeNO) were evaluated at baseline and after four weeks of treatment. Safety was monitored after injection in a clinical setting, as well as via weekly phone follow-up. **Results:** Participants (84.6% male; mean age 67.08 ± 11.42 years) experienced rapid and clinically meaningful improvements at four weeks. CAT score decreased from baseline 21.40 ± 6.22 to 14.00 ± 5.58 (*p* < 0.001) and mMRC scale from 2.90 ± 0.73 to 1.80 ± 0.63 (*p* < 0.0001), respectively. Pre-bronchodilator FEV_1_ increased from baseline 1.35 ± 0.65 L to 1.59 ± 0.84 L (*p* < 0.05), and FVC from 2.36 ± 0.92 L to 2.83 ± 1.11 L (*p* < 0.01). A marked lung deflation was observed: indeed, residual volume declined from baseline 4.17 ± 1.98 L to 3.47 ± 2.07 L (*p* < 0.05), with a concomitant reduction in specific effective airway resistance (from baseline 3.15 ± 1.77 to 2.43 ± 1.44 kPa·s; *p* < 0.05) associated with significant increases in mid-expiratory flow (FEF_25−75_: from baseline 0.62 ± 0.38 to 0.86 ± 0.71 L/s; *p* < 0.05) and peak expiratory flow (3.80 ± 1.40 to 4.48 ± 1.79 L/s; *p* < 0.01). Type 2 inflammatory biomarkers changed as follows: blood eosinophil count fell from baseline 390.0 ± 43.75 to 190.0 ± 65.47 cells/µL (*p* < 0.001); blood basophil number decreased from baseline 37.50 ± 13.89 to 26.25 ± 13.02 cells/µL (*p* < 0.001); plasma fibrinogen lowered from baseline 388.4 ± 54.81 to 334.9 ± 72.36 mg/dL (*p* < 0.01); FeNO levels dropped from baseline 23.95 ± 18.10 to 14.00 ± 2.04 ppb (*p* < 0.0001). Dupilumab was well tolerated, and no treatment-related serious adverse events or discontinuations were detected. **Conclusions:** Within an exploratory context of daily medical activity referring to eosinophilic COPD already treated with maximal inhaled therapy, we found relevant therapeutic effects of a four-week add-on treatment with dupilumab. In particular, our patients manifested rapid improvements in symptoms, airflow limitation, and lung hyperinflation, paralleled by significant decrements of type 2 inflammatory signatures. Such encouraging results were associated with a favorable short-term safety profile. However, larger and longer studies are necessary to corroborate these preliminary findings.

## 1. Introduction

Chronic obstructive pulmonary disease (COPD) remains a major global health issue, characterized by persistent respiratory symptoms and progressive airflow limitation that is not fully reversible [1,2]. As a leading cause of morbidity and mortality worldwide, it imposes a significant and growing burden on patients, healthcare systems, and national economies [3,4]. The clinical presentation of COPD extends beyond simple breathlessness; patients often suffer from chronic cough, sputum production, and debilitating fatigue, which severely impair their quality of life [5,6]. A critical feature of the disease is the occurrence of acute exacerbations, namely episodes of worsening symptoms that frequently require hospitalization, accelerate the long-term decline of lung function, and are associated with a significant increase in mortality risk [7,8]. The pathophysiology of COPD is complex and heterogeneous. While traditionally viewed as a neutrophil-dominant inflammatory disease driven by exposure to noxious particles like cigarette smoke, our understanding has evolved to recognize distinct inflammatory endotypes [9,10]. In a relevant subset of COPD patients, a growing body of evidence highlights the crucial role of type 2 inflammation, characterized by the presence of eosinophils associated with high levels of type 2 cytokines such as interleukin-4 (IL-4), interleukin-5 (IL-5), and interleukin-13 (IL-13) [11,12]. This eosinophilic phenotype, estimated to be present in approximately 20–40% of the COPD population, is linked to a higher frequency of exacerbations and a more rapid disease progression, thereby representing a distinct therapeutic target (treatable trait) [13,14].

Despite recent advances in standard-of-care treatments, including triple therapy with inhaled corticosteroids (ICS), long-acting β_2_-agonists (LABA), and long-acting muscarinic antagonists (LAMA), a substantial proportion of patients with eosinophilic COPD continue to experience frequent exacerbations and a significant symptom burden [15,16]. Therefore, such a clinical context delineates critical unmet needs requiring more targeted treatments that can address the underlying inflammatory drivers of the disease in this specific population [17]. The successful development of biologic therapies focused on type 2 inflammatory mechanisms has revolutionized the management of other chronic respiratory conditions, most notably severe asthma, thus providing a strong rationale for their investigation in COPD [18,19].

Dupilumab is a fully human monoclonal antibody that specifically targets the type 2 inflammatory pathway by blocking the alpha subunit of the IL-4 receptor, thereby inhibiting the signaling network activated by both IL-4 and IL-13, which are key drivers of type 2 inflammation [20,21]. By interrupting this proinflammatory cascade, dupilumab has the potential to prevent multiple downstream effects of IL-4 and IL-13, including eosinophil recruitment, mucus hypersecretion, and airway remodeling, which contribute to the pathophysiology of eosinophilic COPD [22]. The efficacy and safety of dupilumab in this patient population have been robustly demonstrated in large, pivotal, randomized controlled trials (RCTs), such as BOREAS and NOTUS studies [23,24]. These landmark trials showed that, when added to standard triple inhaled therapy in patients with COPD and evidence of type 2 inflammation, dupilumab significantly reduced the annual rate of moderate-to-severe exacerbations and led to clinically meaningful improvements in lung function and health-related quality of life.

While the results from these clinical trials are highly promising, their findings are derived from a carefully selected population under idealized conditions. However, there is a pressing need to understand the effectiveness of dupilumab in a clinical practice setting that includes a broader, more diverse patient population with a wider range of comorbidities, ages, and adherence patterns than those typically selected for a controlled trial [25,26]. Although the long-term durability of pharmacologic actions is a key goal, initial data on the immediate therapeutic impact of dupilumab is essential for clinicians and patients making treatment decisions. Hence, our present exploratory study aimed to investigate the short-term effects of dupilumab on lung function, symptom control, and patient-reported outcomes in a cohort of COPD patients with an eosinophilic phenotype, thus providing an early perspective on its clinical utility.

## 2. Patients and Methods

### 2.1. Study Design and Endpoints

This observational study enrolled consecutive outpatients with eosinophilic COPD receiving add-on biologic treatment with dupilumab between January 2025 and August 2025. All participants were evaluated at the Pulmonology Unit of “Magna Graecia” University Hospital in Catanzaro, Italy, and at the Respiratory Medicine Unit of University of Catania, Italy. This was an exploratory study embedded in a compassionate-use program. No formal a priori sample size calculation was performed; the cohort consisted of all consecutive eligible patients with eosinophilic COPD who initiated dupilumab at the two participating centers during the predefined enrolment period. However, for illustrative purposes we subsequently estimated the sample size that would be required for a similarly designed study using the change in pre-bronchodilator FEV_1_ as a key endpoint. In the phase 3 BOREAS and NOTUS randomized trials, dupilumab added to maximal inhaled therapy increased pre-bronchodilator FEV_1_ at week 12 by approximately 140–160 mL from baseline in the active arms [23,24]. Assuming an expected mean improvement of 150 mL (0.15 L), a standard deviation of change of 200 mL (0.20 L), a two-sided α of 0.05, and 80% power, the standard formula for paired comparisons indicates that about 14 patients would be required. Our final cohort of 13 patients thus approximates the theoretical sample size for a small exploratory study, but remains insufficient for definitive confirmatory conclusions.

Access to dupilumab for the patients recruited in this study was provided through a compassionate use program, in accordance with national regulations for pre-approval drug utilization. Dupilumab was administered subcutaneously at a dose of 300 mg every two weeks. The inclusion criteria were the following: ≥40 years of age, physician diagnosis of COPD with ≥2 moderate or ≥1 severe exacerbations in the last year, modified British Medical Research Council (mMRC) dyspnea scale ≥ 2, blood eosinophils ≥ 300 cells/µL, and current treatment with ICS + LAMA + LABA. We excluded patients with pulmonary diseases other than COPD, cor pulmonale, hypercapnia requiring bilevel ventilation, as well as pregnant or breastfeeding women. This observational study met the standards of Good Clinical Practice (GCP) and the principles of the Declaration of Helsinki, and was authorized by the Ethics Committee of Calabria Region, Italy (Catanzaro, Italy; authorization No. 228/2025).

### 2.2. Data Collection and Assessment

Clinical, functional, and biological data were collected at baseline and after four weeks of add-on treatment with dupilumab. At baseline, we systematically recorded major comorbidities (including arterial hypertension, chronic heart failure and atrial fibrillation). We evaluated both mMRC [27] and COPD Assessment Test (CAT) questionnaires [5], routine hematological tests (including plasma fibrinogen, blood eosinophil levels, blood basophil counts, and total serum IgE), fractional exhaled nitric oxide (FeNO), and lung function. In particular, specific effective resistance (sR_eff_), residual volume (RV), inspiratory capacity (IC), functional residual capacity (FRC), forced vital capacity (FVC), pre-bronchodilator forced expiratory volume in one second (FEV_1_), peak expiratory flow (PEF), and forced expiratory flow between 25% and 75% of FVC (FEF_25−75_) were measured at baseline and after four weeks of treatment with dupilumab. Spirometry and body plethysmography were performed using the MasterScreen pulmonary function testing system and MasterScreen Body (Jaeger-Viasys; CareFusion, Höchberg, Germany), following ATS/ERS guidelines [28]. A Vivatmo Pro FeNO device (Bosch, Waiblingen, Germany) was used to measure FeNO levels according to ATS/ERS standards [29,30].

Following dupilumab administration, patients were monitored for two hours in order to detect any potential adverse effect. Additionally, the emergence of adverse events was evaluated during weekly telephone calls after each treatment.

### 2.3. Statistical Analysis

Normally distributed data were expressed as mean ± standard deviation (SD), while skewed data were reported as median with interquartile range (IQR). The choice of parametric or non-parametric tests depended on data normality, which was evaluated using the Anderson–Darling and Kolmogorov–Smirnov tests. When appropriate, comparisons between variables were made using the Wilcoxon signed-rank test and the paired *t*-test. All analyses were exploratory and limited to paired comparisons between baseline and week 4 within the same subjects; no formal adjustment for multiple testing was applied. A *p*-value below 0.05 (two-tailed) was considered as statistically significant. All statistical analyses and figures were generated using Prism Version 10.3.0 (GraphPad Software Inc., San Diego, CA, USA).

## 3. Results

Among the 13 eosinophilic COPD patients included in this study, 11 (84.62%) were male. The mean age was 67.08 ± 11.42 years, and the mean disease duration was 15.63 ± 9.95 years. The median body mass index (BMI) at baseline was 26.63 kg/m^2^ (23.65–27.33), and all patients were current smokers or ex-smokers. The baseline characteristics of enrolled patients are summarized in Table 1. Only 2 out of 13 (15.39%) showed evidence of sensitization to common aeroallergens.

**Figure 1:**
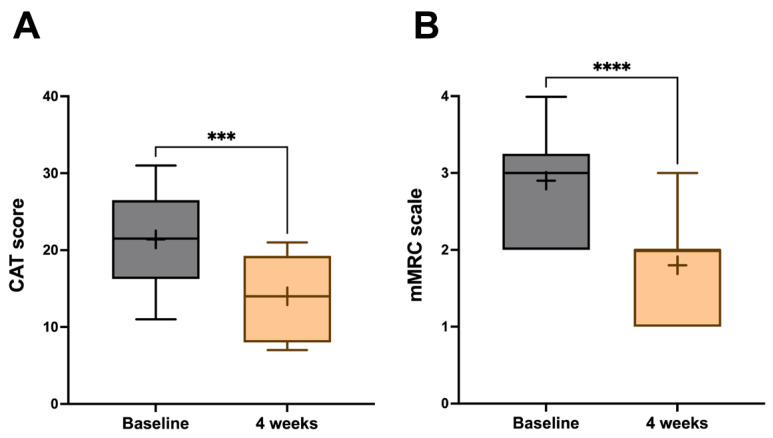
Efficacy of dupilumab with regard to CAT score (**A**) and mMRC scale (**B**). The ‘+’ symbol is plotted at the mean. The line in the middle of the box indicates the median value; the box extends from the 25th to 75th percentile. Whiskers express the highest and the lowest values. Abbreviations: *** *p* < 0.001; **** *p* < 0.0001.

After four weeks of add-on therapy with dupilumab, the CAT score significantly decreased from a baseline value of 21.40 ± 6.22 to 14.00 ± 5.58 (*p* < 0.001) (Figure 1A), and mMRC changed from 2.90 ± 0.73 to 1.80 ± 0.63 (*p* < 0.0001) (Figure 1B), thus indicating improved symptom control.

Before starting dupilumab, patients had been on triple inhaled therapy for a median of 14 months (12–17). The clinical effects of dupilumab were matched by notable improvements in lung function (Table 2). After four weeks of add-on biological therapy, pre-bronchodilator FEV_1_ increased from baseline 1.35 ± 0.65 L to 1.59 ± 0.84 L (*p* < 0.05), while FVC rose from 2.36 ± 0.92 L to 2.83 ± 1.11 L (*p* < 0.01). One month after the initial dupilumab injection, the average sR_eff_ decreased from 3.15 ± 1.77 kPa·s to 2.43 ± 1.44 kPa·s (*p* < 0.05). Dupilumab induced a significant effect on lung hyperinflation. Indeed, RV decreased in 11 (84.62%) out of our 13 patients, dropping down from baseline 4.17 ± 1.98 L to 3.47 ± 2.07 L (*p* < 0.05). This RV reduction was paralleled by relevant improvements in FEF_25−75_, which increased from 0.62 ± 0.38 L/s to 0.86 ± 0.71 L/s (*p* < 0.05). PEF changed from 3.80 ± 1.40 L/s to 4.48 ± 1.79 L/s (*p* < 0.01).

**Figure 2:**
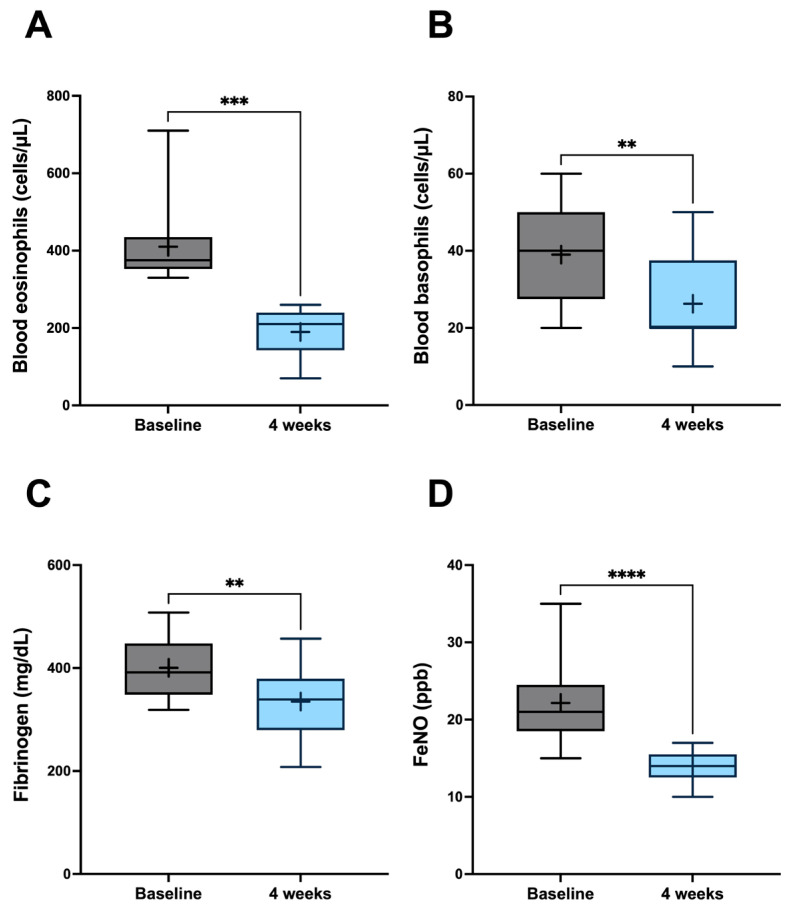
Efficacy of dupilumab with regard to blood eosinophils (**A**), blood basophils (**B**), plasma fibrinogen (**C**), and FeNO (**D**). The ‘+’ symbol is plotted at the mean. The line in the middle of the box indicates the median value; the box tends from the 25th to 75th percentile. Whiskers express the highest and the lowest values. Abbreviations: ** *p* < 0.01; *** *p* < 0.001; **** *p* < 0.0001.

After starting add-on biological treatment, blood eosinophil levels significantly decreased from a baseline count of 390.0 ± 43.75 cells/µL to 190.0 ± 65.47 cells/µL (*p* < 0.001) (Figure 2A), and blood basophil numbers dropped from 37.50 ± 13.89 cells/µL to 26.25 ± 13.02 cells/µL (*p* < 0.001) (Figure 2B). Moreover, during the same period, plasma fibrinogen decreased from 388.4 ± 54.81 mg/dL to 334.9 ± 72.36 mg/dL (*p* < 0.01) (Figure 2C), and FeNO levels fell from a baseline value of 23.95 ± 18.10 to 14.00 ± 2.04 ppb (*p* < 0.0001) (Figure 2D). Total serum IgE levels did not significantly change, persisting at 23.90 IU/mL (14.68–585.9) and 22.95 IU/mL (12.10–498.2) (*p* = 0.190) at baseline and after four weeks of dupilumab treatment, respectively.

The safety profile of dupilumab was very satisfactory in this cohort. As an additional biological therapy, dupilumab was well tolerated and did not trigger any treatment-related serious adverse event during the observation period. Discontinuations due to adverse events did not occur, and no patient skipped any administration. Mild events were rare and self-limiting, mainly consisting of sporadic injection-site reactions. Eosinophil-related complications were not detected at all, and no cases of conjunctivitis were observed.

## 4. Discussion

Within the context of our daily medical activity, this investigation provides timely and practical evidence that add-on dupilumab can deliver rapid, clinically meaningful benefits for patients with severe, frequently exacerbating eosinophilic COPD who remain highly symptomatic despite optimized triple inhaled therapy. Within just four weeks, we observed significant improvements across the key areas of daily practice. Indeed, patients reported less dyspnea and a better health status, spirometry showed early relief of airflow limitation, and body plethysmography demonstrated notable lung deflation, evidenced by a significant reduction in residual volume. These changes were paralleled by swift decreases in type 2 inflammatory biomarkers, thereby suggesting an alignment of mechanistic signals with clinical outcomes, and also indicating that the IL-4/IL-13-driven eosinophilic endotype of COPD can be rapidly reversible.

Our findings should be interpreted within the evidence context established by both BOREAS and NOTUS randomized controlled trials, which showed that dupilumab decreased the annual exacerbation rate and improved lung function over 52 weeks in carefully selected, type-2-high COPD patients [23,24]. While RCTs have high internal validity, they inevitably operate under strict eligibility criteria and controlled conditions that may limit their wider applicability [31]. The similar pattern of benefit observed in RCTs supports the real-world effectiveness of dupilumab. Importantly, we demonstrate that meaningful improvements can occur within one month, which is clinically significant for reinforcing adherence, guiding early treatment decisions, and setting realistic expectations for symptomatic relief, especially regarding dyspnea and its impact on daily life [32]. In our cohort, the mean CAT score decreased by 7.4 points after four weeks, well beyond the established MCID of two points, indicating a significant improvement in perceived health status and daily functioning [33]. At the same time, dyspnea on the mMRC scale improved substantially, shifting for many patients from a level at which they needed to stop after short walks to one where they could carry out routine tasks without severe breathlessness. These rapid and substantial symptomatic improvements strongly suggest that dupilumab quickly attenuates the mechanical and sensory drivers of dyspnea, such as mucosal edema, mucus hypersecretion, and small-airway hyperresponsiveness, that are closely linked to the IL-13 axis and can increase the inspiratory load via dynamic lung hyperinflation [34]. When airway narrowing eases and air trapping diminishes, inspiratory muscles work at more optimal length–tension relationships, and breathlessness lessens in a way that patients notice immediately [35].

Functional results reflected the patient-reported improvements. Over the same four-week period, mean pre-bronchodilator FEV_1_ increased by more than 200 mL. For an early time point, this is noteworthy in absolute terms and compares favorably with the 12-week FEV_1_ difference reported versus placebo in NOTUS [24]. Mid-expiratory flow also improved, as shown by FEF_25−75_ increase, consistent with enlargement of small airways, often disproportionately affected in type 2-high disease. The most significant physiological change is probably the marked reduction in residual volume—about 700 mL—over just one month. Indeed, in COPD, lung hyperinflation is the main pathophysiologic nexus linking expiratory flow limitation to dyspnea and exercise intolerance [36]. Achieving such a degree of lung deflation over weeks rather than months is uncommon in advanced COPD and suggests that, in this eosinophilic endotype, a substantial component of airflow limitation and premature airway closure is dynamic and rapidly reversible with IL-4/IL-13 pathway blockade. Although this rapid reduction is striking, the absence of a control group prevents us from ruling out alternative explanations such as optimization of inhaler technique, learning effects during repeated testing, or regression to the mean. Biomarker dynamics further support a targeted mechanism. All patients were chosen with baseline blood eosinophils ≥ 300 cells/µL, and systemic eosinophil count decreased after treatment, reinforcing the idea that blood eosinophilia indicates responsiveness to type 2-targeted therapy [37]. FeNO reduction indicates early suppression of IL-13-driven airway inflammation, whereas total serum IgE remained largely unchanged, in line with its slower kinetics after IL-4Rα blockade [38]. The rapid decrease in plasma fibrinogen was also noteworthy. Elevated fibrinogen levels reflect a systemic inflammatory tone and are linked to higher exacerbation risk, cardiovascular morbidity, and all-cause mortality in COPD patients [39,40,41]. The high baseline levels of fibrinogen detected in our cohort marked a population at increased systemic risk; the quick therapeutic response to dupilumab suggests that the IL-4/IL-13 cytokine axis may contribute not only to airway pathophysiology, but also to the extra-pulmonary inflammatory burden characterizing this type 2 endotype. Nonetheless, our observation of improvement in symptom burden and lung function after four weeks is consistent with emerging real-world data in COPD patients with type 2 inflammation treated with dupilumab, in whom a marked reduction in exacerbations and improvement in patient-reported outcomes were documented over longer follow-up [42].

Safety and tolerability in the elderly, comorbid COPD population are of crucial importance. In our experience, dupilumab was well tolerated over the four-week observation period. No treatment-related serious adverse events occurred, no discontinuations or interruptions were necessary, and mild events were infrequent, transient, and mostly limited to sporadic injection-site reactions. We found no eosinophil-related complications and no cases of conjunctivitis or dry-eye disease. Longer observation will be required to detect rarer or delayed adverse events, but these early data offer reassurance for clinicians considering dupilumab use in routine care.

The strengths of the current study enhance its relevance to everyday practice. We focused on a high-need, difficult-to-treat population with severe eosinophilic COPD who continue to experience symptoms and exacerbations despite maximized inhaled therapy, thereby providing insights that are immediately useful for decision-making at the point of care. The inclusion of body plethysmography adds a detail rarely seen in randomized trials, showing that early benefits go beyond FEV_1_ changes, thus including significant relief of lung hyperinflation. Additionally, the combination of multiple biomarkers—eosinophils, FeNO, basophils, and fibrinogen—offers convergent mechanistic support that reinforces the internal consistency of clinical and physiological findings.

Nonetheless, limitations should influence interpretation. This was a small population without a concurrent control group, so that effects like regression to the mean, placebo-like responses on patient-reported outcomes, and natural variability cannot be fully ruled out. The actual physiological change—especially the 700 mL reduction in RV—makes a purely subjective explanation unlikely, though confounding effects remain possible. In particular, RV decreased in 11 (84.62%) out of our 13 patients. This reduction was really striking in 6 (46.15%) out of the 13 enrolled participants. Of course, given the small number of treated patients, the very relevant RV reduction detected in almost half of the subjects probably magnified the overall effect. However, the very relevant RV decrease is consistent with the significant FEF_25−75_ increase, as well as with the changing trends in IC and FRC. Indeed, it can be reasonably argued that dupilumab was able to markedly improve eosinophilic inflammation within the small airways of our COPD patients. As a consequence, the greater expiratory airflow could explain the reduction in air-trapping and RV.

The brief four-week timeframe prevents any firm conclusion about the durability of benefits, long-term safety, or impacts on exacerbations, which are key factors in morbidity, mortality, and social/economic costs of COPD. The small sample size, absence of an external control group, and limited follow-up prevent making definitive conclusions about long-term effectiveness, response durability, reduction in exacerbation risk, or safety. The improvements observed in symptoms, lung volumes, and systemic biomarkers should therefore be considered as early indicators of response that need confirmation and further investigation through larger cohorts with extended follow-up and proper control of multiplicity. Thus, such considerations hinder the generalizability of our conclusions. Finally, the restricted two-center, compassionate-use setting might introduce selection biases and limit broader applicability. Therefore, confirming these results across multiple centers with standardized protocols will be essential. Looking ahead, this research field would benefit from larger, longer-term investigations that could confirm the durability of symptomatic and physiological improvements, as well as quantify the reductions in exacerbations, hospitalizations, and oral corticosteroid exposure over twelve months and beyond. In conclusion, among patients with severe eosinophilic COPD already receiving maximal inhaled therapy, dupilumab produced rapid and consistent beneficial effects within four weeks, encompassing symptoms, airflow limitation, and—most importantly—lung hyperinflation, associated with simultaneous decreases in blood eosinophils/basophils, FeNO, and fibrinogen that indicate an on-target mechanism. Relief of pulmonary hyperinflation appears to be central for the early clinical effects of IL-4/IL-13 receptor blockade in this type 2 COPD endotype, and warrants specific focus on both functional assessment and patient communication. However, the small sample size and the lack of a control arm mean that it may not be possible to generalize our conclusions. Despite these important limitations, we think that our real-world observations maintain a reliable relevance, based on the relevant improvement of symptoms, as well as on the clinical significance of lung function changes. The next research steps should clarify how enduring these improvements are, measure their impact on exacerbations and survival-related outcomes, and determine whether rapid systemic anti-inflammatory effects indicate significant reductions in cardiometabolic risk.

## Figures and Tables

**Table 1:** Patient characteristics.

Characteristic	Baseline	Follow-Up	*p* Value
Age, mean ± SD, years	67.08 ± 11.42	67.15 ± 11.47	0.337
Male gender, N (%)	11 (84.62)	11 (84.62)	>0.99
Weight, mean ± SD, kg	72.36 ± 9.54	72.18 ± 9.44	0.167
Height, mean ± SD, cm	168.72 ± 10.50	168.72 ± 10.50	>0.99
BMI, median (IQR), kg/m^2^	26.63 (23.65–27.33)	26.54 (23.65–27.33)	0.169
Smokers and ex-smokers, N (%)	13 (100.00)	13 (100.00)	>0.99
Arterial hypertension, N (%)	7 (53.85)	7 (53.85)	>0.99
Heart failure, N (%)	2 (15.38)	2 (15.38)	>0.99
Atrial fibrillation, N (%)	3 (23.08)	3 (23.08)	>0.99
CAT score, mean ± SD	21.40 ± 6.22	14.00 ± 5.58	<0.001
mMRC scale, mean ± SD	2.90 ± 0.73	1.80 ± 0.63	<0.0001
Blood eosinophils, mean ± SD, cells/µL	390.0 ± 43.75	190.0 ± 65.47	<0.001
FeNO, mean ± SD, ppb	23.95 ± 18.10	14.00 ± 2.04	<0.0001
IgE, median (IQR), IU/mL	23.90 (14.68–585.9)	22.95 (12.10–498.2)	0.190

Abbreviations. CAT, COPD Assessment Test; mMRC, modified British Medical Research Council; FeNO, fractional exhaled nitric oxide; SD, standard deviation; IQR, interquartile range.

**Table 2:** Lung function parameters before and after treatment with dupilumab.

Lung Function Parameter	Baseline	Follow-Up	*p* Value
FEV_1_, mean ± SD, % pred.	46.08 ± 17.33	53.75 ± 20.76	<0.001
FEV_1_, mean ± SD, L	1.35 ± 0.65	1.59 ± 0.84	<0.05
FVC, mean ± SD, % pred.	62.08 ± 15.71	74.08 ± 18.44	<0.001
FVC, mean ± SD, L	2.36 ± 0.92	2.83 ± 1.11	<0.01
sR_eff_, mean ± SD, % pred.	275.80 ± 145.00	210.30 ± 121.50	<0.05
sR_eff_, mean ± SD, kPa·s	3.15 ± 1.77	2.43 ± 1.44	<0.05
RV, mean ± SD, % pred.	175.00 ± 90.09	146.50 ± 93.80	<0.05
RV, mean ± SD, L	4.17 ± 1.98	3.47 ± 2.07	<0.05
IC, mean ± SD, % pred.	53.10 ± 17.68	61.20 ± 29.00	0.177
IC, mean ± SD, L	1.41 ± 0.51	1.63 ± 0.71	0.163
FRC, mean ± SD, % pred.	156.30 ± 59.36	146.80 ± 59.21	0.056
FRC, mean ± SD, L	5.34 ± 2.08	5.01 ± 2.07	0.059
FEF_25−75_, mean ± SD, % pred.	27.17 ± 15.47	35.75 ± 22.94	<0.05
FEF_25−75_, mean ± SD, L/s	0.62 ± 0.38	0.86 ± 0.71	<0.05
PEF, mean ± SD, % pred.	54.90 ± 18.19	63.30 ± 20.14	<0.01
PEF, mean ± SD, L/s	3.80 ± 1.40	4.48 ± 1.79	<0.01

Abbreviations. FEV_1_, forced expiratory volume in one second; FVC, forced vital capacity; sR_eff_, specific effective resistance; RV, residual volume; IC, inspiratory capacity; FRC, functional residual capacity; FEF_25−75_, forced expiratory flow between 25% and 75% of FVC; PEF, peak expiratory flow; SD, standard deviation.

## Data Availability

The authors will share all individual participant data collected during the trial, after de-identification, with researchers who provide a methodologically sound proposal.
